# A scalable neuroinformatics data flow for electrophysiological signals using MapReduce

**DOI:** 10.3389/fninf.2015.00004

**Published:** 2015-03-16

**Authors:** Catherine Jayapandian, Annan Wei, Priya Ramesh, Bilal Zonjy, Samden D. Lhatoo, Kenneth Loparo, Guo-Qiang Zhang, Satya S. Sahoo

**Affiliations:** ^1^Division of Medical Informatics, School of Medicine, Case Western Reserve UniversityCleveland, OH, USA; ^2^Department of Electrical Engineering and Computer Science, School of Engineering, Case Western Reserve UniversityCleveland, OH, USA; ^3^Department of Neurology, School of Medicine, Case Western Reserve UniversityCleveland, OH, USA

**Keywords:** electrophysiological signal data, epilepsy research, MapReduce, cloudwave signal format, epilepsy and seizure ontology

## Abstract

Data-driven neuroscience research is providing new insights in progression of neurological disorders and supporting the development of improved treatment approaches. However, the volume, velocity, and variety of neuroscience data generated from sophisticated recording instruments and acquisition methods have exacerbated the limited scalability of existing neuroinformatics tools. This makes it difficult for neuroscience researchers to effectively leverage the growing multi-modal neuroscience data to advance research in serious neurological disorders, such as epilepsy. We describe the development of the Cloudwave data flow that uses new data partitioning techniques to store and analyze electrophysiological signal in distributed computing infrastructure. The Cloudwave data flow uses MapReduce parallel programming algorithm to implement an integrated signal data processing pipeline that scales with large volume of data generated at high velocity. Using an epilepsy domain ontology together with an epilepsy focused extensible data representation format called Cloudwave Signal Format (CSF), the data flow addresses the challenge of data heterogeneity and is interoperable with existing neuroinformatics data representation formats, such as HDF5. The scalability of the Cloudwave data flow is evaluated using a 30-node cluster installed with the open source Hadoop software stack. The results demonstrate that the Cloudwave data flow can process increasing volume of signal data by leveraging Hadoop Data Nodes to reduce the total data processing time. The Cloudwave data flow is a template for developing highly scalable neuroscience data processing pipelines using MapReduce algorithms to support a variety of user applications.

## Introduction

Electrophysiological signal data, such as electroencephalogram (EEG) and electrocardiogram (ECG), are critical to both neuroscience research and patient care (Bartolomei et al., 2008; Wendling et al., 2010). For example, EEG is recorded using electrodes placed on the surface or inside the brain to record electrical activity, which include detection of seizure events, location of seizure, and seizure signal characteristics. EEG signal data plays a key role in neurological disease treatment, for example it is used as gold standard for identifying the seizure onset zone in focal epilepsy patients during presurgical evaluation (Rosenow and Lüders, 2001). Epilepsy is the most common serious neurological disorder affecting 65 million persons worldwide with about 150,000 new cases diagnosed each year in the United States alone (Epilepsy Foundation, 2014). EEG data is used to identify the specific brain region that can be removed to reduce or eliminate seizure occurrences. In addition to epilepsy, electrophysiological signal data is also used in sleep and other neurological disorder research (Redline et al., 2013). The growing sophistication of signal recording hardware and signal analysis techniques, for example development of epileptogenicity index using Stereotactic EEG and MRI data for characterizing seizure onset zone (Bartolomei et al., 2008), has significantly increased data management challenges for signal data. The International Neuroinformatics Coordinating Facility (INCF) aims to address some of these data management challenges, including development of common data representation format and use of consistent terminological system, through collaborative initiatives (INCF). However, the existing neuroinformatics software tools have limited capability to address these challenges and do not scale with increasing volume of signal data to support user requirements (Schlögl, 2010).

For example, epilepsy patients are typically admitted for a five-day period in an epilepsy monitoring unit (EMU) to record electrophysiological signals from multiple channels, which generates about 10–20 gigabytes (GB) of signal data. The signal data is analyzed by epileptologists using standalone signal visualization and analysis tools to detect clinical events, for example start or end of seizures, changes in heart rate, and signal characteristics during a seizure event (Lüders et al., 2012). These manually identified clinical events are stored in a separate text annotation file, whereas the signal data is usually stored using the European Data Format (EDF; Kemp and Olivan, 2003). EDF is a de-facto standard for storing signal data with associated metadata, such as recording details (duration of a data record, transducer type) and the study information (patient description), in the epilepsy community. However, EDF files are not suitable for fast access to random segments of signal data in response to user queries, combining data from different channels to compose a signal montage, and efficient network transfer to remote user applications (e.g., signal visualization). In addition, the separate storage of clinical event annotations makes it difficult to ensure synchronized changes with signal data, coordinated data transfer, and integration with user applications (Schlögl, 2010).

Existing signal data management approaches use multiple software programs and data processing scripts that: (a) require manual intervention at each step; (b) are difficult to maintain and re-use across projects; and (c) have significant limitations in terms of scalability as well as efficiency. For example, it takes approximately 8 h to process a single EDF file using existing signal processing tools and about 3–4 days to process all signal data recorded during a single patient visit to the EMU. The limitations of existing data processing tools are exacerbated by the increasing volume of signal data collected by sophisticated techniques, for example use of intracranial electrodes to record signals at a high resolution. The large *volume* and high *velocity* (rate of data generation as well as need for fast analysis) clearly identify signal data as an example of “clinical Big Data” (Agrawal et al., 2012). For example, the EMU at the Case Western Reserve University Neurology Department has generated 20 terabytes (TB) of data in the past 3 years and the rate of data collection is increasing each year. This requires the development of highly scalable signal processing and storage techniques using distributed and parallel computing infrastructure that can keep pace with signal Big Data.

In addition to the volume and velocity of signal data, there is a clear need to address the challenge of *variety* in signal data, which is often generated at different sites using disparate recording protocols. The use of heterogeneous terms to describe clinical events and signal metadata also make it difficult to ensure consistent interpretation of signal data annotations and support data sharing or integration. Consistent use of terminology is specifically important in the epilepsy community due to the well-known challenges in epilepsy classification with its inherent complexity and requirements of different stakeholders (Berg et al., 2010; Lüders et al., 2012). The role of well-defined terminological system has also been highlighted to enhance the secondary use of biomedical data (Holdren and Lander, 2010). A common terminological system modeled using formal knowledge representation language, for example domain ontologies, will support easier data sharing and development of re-usable neuroinformatics tools.

### Related Work

The existing work on electrophysiological signal data management can be divided into two categories: (a) Data Representation Formats; and (b) Data Processing Tools. Although there is no existing standard for signal data representation, there are a large number of data formats developed by instrument vendors, researchers, and different neuroscience projects (Schlögl, 2010; Sobolev et al., 2014a). Signal data representation formats need to meet the requirements of multiple stakeholders and address multiple challenges, including the inherent complexity of signal data such as different sampling rates and scaling factors (Schlögl, 2010). The General Data Format (GDF), which is part of the BioSig platform (Vidaurre et al., 2011), was proposed to meet several of these requirements, such as representing all physical units of the signal and multiple binary data types (Schlögl, 2006). Similarly, the Neuroscience Electrophysiology Object (NEO) is a well-known object-oriented data representation format with extensive Application Program Interface (API) support implemented in the Python programming language (Garcia et al., 2014). The NEO format supports representation of both signal metadata, for example sampling intervals and brain location for signal recording together with the signal data.

The German Neuroinformatics Node (G-Node) integrates the NEO format with the open metadata Markup Language (odML; Grewe et al., 2011) to define the GNData format for use in a data management platform (Sobolev et al., 2014b). In addition to the data format, the GNData signal data management platform aims to develop a common storage layer with a generic API based on Representation State Transfer (REST) web services for signal data annotation and access control (Sobolev et al., 2014a). The GNData platform also uses the Hierarchical Data Format (HDF5) (Hierarchical Data Format (HDF5), 2014), which has generated a lot of interest in the neuroinformatics community as a potential common representation standard, to store the signal data. The INCF dataspace is a cloud-based signal data storage platform that supports exchange and storage of signal data using the Integrated Rule-Oriented Data System (iRODS) software (INCF International Neuroinformatics Coordination Facility (INCF), 2014). The Carmen project has developed a workflow tool to support analysis of neuroscience data in a virtual laboratory using a library of services and tools based on the Neurophysiology Data translation Format (NDF; Weeks et al., 2013). The Neuroscience Information Framework (NIF) has created an ontology-based resource describing many neuroscience terms, for example diseases and brain anatomy, which can be used as starting point for reconciling terminological heterogeneity (Imam et al., 2012).

Some recent projects have identified the need to develop a neuroscience domain ontology to standardize the terminology used for signal data annotation (Mouček et al., 2014). Ontologies are widely used as reference terminology in the biomedical community to standardize terms and support knowledge discovery over ontology-annotated data (Ashburner et al., 2000; The National Center for Biomedical Ontology, 2014). However, the proposed Ontology for Experimental Neuroscience (OEN) is not publicly available for review and evaluation at present. In addition, there is no available documentation demonstrating the use of OEN in any existing project, including the EEG/ERP portal (Mouček et al., 2014). At present, we are not aware of any existing work that uses an ontology-based scalable computing approach to develop an integrated data flow for signal processing, which addresses the three challenges of volume, velocity, and variety in signal data management. We describe the *Cloudwave data flow* in this paper that aims to address these challenges by using a MapReduce-based signal processing algorithm together with an epilepsy domain ontology for signal data annotation.

### Cloudwave Project: Managing Electrophysiological Signal Big Data

The Cloudwave project is being developed as part of a multi-center epilepsy research project to study the potential biomarkers of sudden unexpected death in epilepsy (SUDEP; Lhatoo, 2014) The three primary aims of the Cloudwave project are to: (a) develop scalable neuroinformatics data processing and storage approaches using parallel programming over distributed computing infrastructure; (b) use domain ontology together with flexible data representation format for data integration and analysis; and (c) develop a Web browser-based visualization and query interface to support multi-center collaborative research. The Center for SUDEP Research (CSR) has been funded by the U.S. National Institutes of Neurological Disorders and Stroke (NINDS), which brings together domain expertize in human and animal models of epilepsy to advance SUDEP research (Lhatoo, 2014). The CSR builds on the earlier Prevention and Risk Identification of SUDEP Mortality (PRISM) project, which involved EMUs at the University Hospitals Case Western Reserve University (CWRU), the Ronald Reagan Medical Center (University of California, Los Angeles), the Northwestern Memorial Hospital (Chicago), and the National Hospital for Neurology and Neurosurgery (London, UK) (Lhatoo, 2011).

In earlier work, we have described the development of the Cloudwave signal visualization user interface (Jayapandian et al., 2013b) and initial results from using parallel computing approach to extract channel-specific signal data from EDF files (Jayapandian et al., 2013a). In this paper, we describe:

1. Development and evaluation of an integrated signal data processing pipeline implemented using MapReduce programming approach to support user application, such as signal visualization;
2. Define a flexible signal data partitioning technique that support processing and transfer of large volume of signal data in a distributed computing environment; and
3. Development of a new epilepsy-focused Cloudwave Signal Format (CSF) that uses domain ontology for signal data annotation and is compatible with existing representation formats, such as HDF5 and NEO.

The rest of the paper is organized as follows: Section Material and methods describes the components of the Cloudwave data flow, including data partitioning technique, MapReduce algorithm, the CSF, and the epilepsy domain ontology. Section Results describes the results of our evaluation that demonstrates the flexibility of the Cloudwave data partitioning technique and scalability of the data flow. Section Discussion discusses the applicability of the Cloudwave data flow in existing neuroscience data management projects and the wider neuroinformatics community followed by conclusion in Section Conclusion.

## Material and Methods

**Figure 1:**
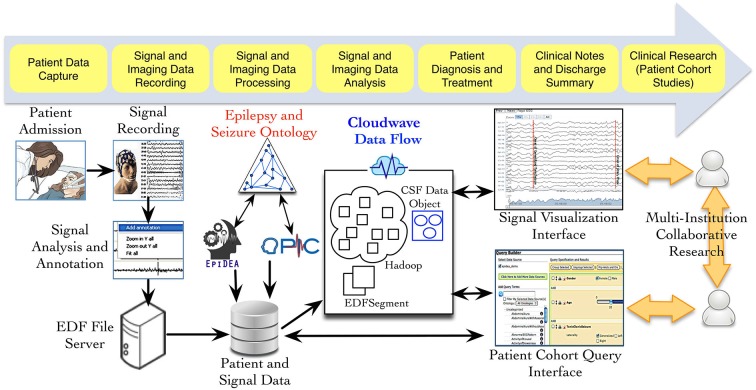
**Data acquisition and management in Epilepsy Monitoring Unit (EMU)**. Multiple modalities of data are generated during patient stay in an EMU, including electrophysiological signal data. Three neuroinformatics tools have been developed as part of the PRISM project: (a) OPIC for patient information collection, (b) EpiDEA for clinical text processing of discharge summaries and related documents; and (c) Cloudwave for managing signal data. The Cloudwave data flow uses MapReduce and distributed file system to store and process signal data for scalability. The data processed and generated from the Cloudwave data flow is consumed by a Web browser-based signal visualization interface.

Figure 1 provides an overview of the different phases of neuroscience data generation and management in an EMU, which involves data acquisition, storage, and analysis using multiple informatics tools. As part of the PRISM project, we have developed an ontology-driven patient information capture system called OPIC (Sahoo et al., 2012) and a clinical text-processing tool for clinical documents called EpiDEA (Cui et al., 2012). The Cloudwave project is complementary to these tools and aims to process EDF files into self-descriptive objects, which can be stored in a high performance distributed file system and support fast access to random segments of signal data. The data flow is initiated after a user deposits one or more EDF file in a specified folder location, which is regularly polled by a “server process”. The second phase of the data flow partitions the signal data into smaller fragment that is used by the MapReduce algorithm to generate CSF data objects. The CSF data objects consist of signal data, metadata, and epilepsy domain ontology-based signal annotations. In the final phase, CSF data objects are stored in a high performance distributed file system that supports different user applications, such as the Cloudwave signal visualization interface.

### Scalable Electrophysiological Signal Data Processing Using MapReduce

MapReduce is a well-known and widely used parallel programming approach for large-scale data processing and analytical tasks in Web search engines and scientific data processing (Dean and Ghemawat, 2010). The MapReduce approach uses a simple two-step programming model consisting of the “map” and “reduce” functions for data processing and aggregation respectively. A map function generates a set of <*key*, *value*> pairs for each input data record, which are grouped into output records based on a common key. A partition function assigns each output record with common key to a reducer function that aggregates all values with common key and generates the final output record. MapReduce algorithms are usually implemented with multiple map and reduce functions that are executed on different computing nodes. A shuffle function transfers the output records from map functions to appropriate reduce functions based on the mapping of keys to reducers by the partition function (Dean and Ghemawat, 2010).

This two-step programming approach can be generalized to process any type of data and it can be executed multiple times for multi-step data processing workflows. MapReduce algorithms are usually implemented over distributed file system, such as the open source Hadoop Distributed File System (HDFS; Shvachko et al., 2010). HDFS is a high performance file system deployed in a distributed computing platform that uses data replication and parallel file operations to support reliable storage and fast access to large volumes of data. In contrast to traditional desktop file system, HDFS is designed to manage large volumes of heterogeneous data and it can easily scale with increasing volume of data by adding new computing nodes as required. The Cloudwave data flow leverages these features of the open source Hadoop technology stack to efficiently process large volumes of signal data and support reliable storage.

#### Signal Data Processing and Flexible Data Partitioning Technique

**Figure 2:**
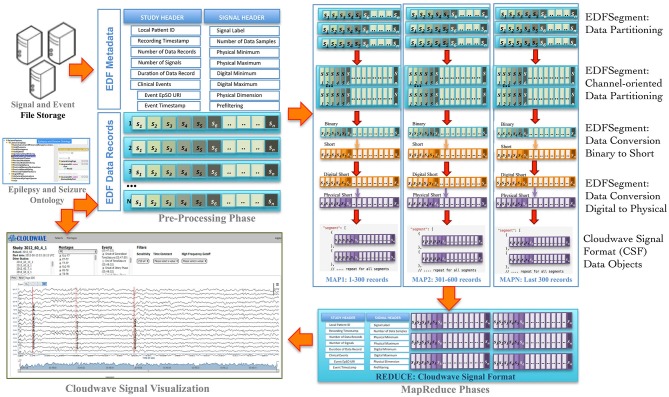
**Cloudwave data flow**. EDF files generated by signal recording instruments are deposited in pre-specified folder, which is regularly polled by a daemon process. If one or more EDF files are detected, the Cloudwave data flow follows multiple steps: (1) in the pre-processing phase signal data is partitioned into *fragments* of specific time duration (*epoch*) and stored in a new self-descriptive structure called EDFSegment, (2) in the second phase, an EDFSegment method is invoked to store signal data in channel-oriented order for easier composition into signal *montages*, (3) in the third phase, the signal data are converted from binary to short integer format and from digital to physical values for use by the Cloudwave signal visualization interface, (4) in the third phase, the EDFSegments are transformed in the Cloudwave Signal Format (CSF) data objects, which are aggregated based on original EDF file identifier in the last phase. The CSF data objects can be efficiently transferred over the network to the Cloudwave signal visualization module as compared to the original EDF files.

Signal visualization applications usually extract and render signal data from a single channel or group of channels, which constitutes a signal montage. For example, six standard montages (M1 to M6) are used for epilepsy signal data analysis. However, an EDF file stores signal data in contiguous set of samples recorded from all channels in a given session (also called EDF *Data Record*), which makes it difficult to access random signal fragments and extract data for specific channel or montage-specific channel data. In addition, signal analysis and visualization applications often require integrated access to clinical event annotations, which are stored separately from the EDF file. The Cloudwave data flow addresses these challenges as well as supports the use of ontology-based annotation by implementing data pre-processing, partitioning, and transformation steps (Figure 2 illustrates the Coudwave data flow). In the first step, the data flow extracts and integrates the signal metadata with clinical event annotation into a single data object. In the next step, the signal data corresponding to each recording channel is extracted from EDF files and integrated into a channel-specific signal data fragments. The data flow transforms these data fragments into CSF with mappings between the clinical event annotation and terms modeled in the epilepsy domain ontology.

In the final step, the data flow converts the signal data stored in binary format to short integer and from “digital values” (generated by analog to digital signal converter) to “physical values” (physiological values) to meet the requirements of the Cloudwave signal visualization interface. These data flow steps are parallelized using MapReduce programming approach. An initial implementation of the data flow used a single EDF file as input to the MapReduce algorithm, which could not be processed on the CWRU Hadoop cluster due to lack of adequate memory in the computing nodes. A Hadoop cluster consists of a single *Master Node* and multiple *Data Nodes*, which execute the computational tasks in a MapReduce algorithm (based on a master-slave configuration). The large volume of signal data in an EDF file (about 1 GB) exhausted the available memory on individual Data Nodes leading to memory error. The Hadoop Java API allows partitioning the input data into smaller sized datasets, which can be distributed and processed in multiple Hadoop Data Nodes. However, there is no existing technique to partition EDF files, which requires partitioning the signal data into appropriate sized *fragments* with the associated signal metadata and clinical event annotations.

**Figure 3:**
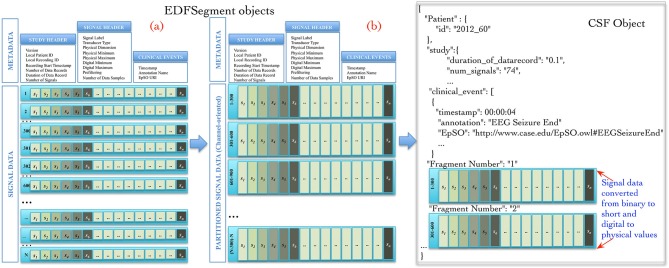
**EDFSegment and CSF object**. During the pre-processing phase, the signal data from study metadata, channel-specific metadata from EDF file is integrated with clinical event annotations and stored with partitioned signal data (fragments corresponding to 30 s epochs). The total number of fragments per EDFSegment is a configurable parameter in Cloudwave data flow that can be adjusted according to available memory in the Hadoop Data Data Nodes. In the first phase of the MapReduce algorithm, the signal data stored as EDF Data Records are transformed into channel oriented data. After additional data processing steps to support the Cloudwave signal visualization module, the CSF data objects are created using the signal data partitioning scheme of the EDFSegments.

The Cloudwave data flow implemented a new “EDFSegment” data structure to address this requirement. An EDFSegment object (Figure 3) consists of the clinical event annotations, study metadata, and metadata corresponding to each channel together with the *fragments* of signal data. Each fragment of signal data corresponds to a single “epoch” of specific time duration, which is a configurable parameter in Cloudwave data flow (30 s is the default duration). A signal fragment consists of multiple EDF Data Records (a Data Record is usually of 0.1 s duration Kemp and Olivan, 2003). The number of signal fragments in a single EDFSegment object is also a configurable parameter in Cloudwave, which is specified according to the available memory resources on individual Hadoop Data Nodes. The EDF Segment objects are generated from EDF files during the pre-processing phase, which allows the Cloudwave data flow to flexibly change the volume of data assigned to each Data Node for successful execution of the MapReduce algorithm.

#### MapReduce Algorithm for Processing Signal Data

The map and reduce functions require input data to be structured as <*key*, *value*> pairs. The Cloudwave data flow generates a unique *key* for each EDFSegment object based on the file identifier and the fragment identifier with the EDFSegment object as *value*. The map function implements the data processing steps in the Cloudwave data flow over multiple Hadoop Data Nodes, which have one or more EDFSegment objects. The EDFSegment object keeps track of the EDF Data Records, the order of signal fragments, and the order of channel recording, which is converted into structural metadata in the CSF data objects. The Cloudwave data flow uses the EDFSegment object to store and transfer signal data across intermediate processing steps (Figure 3 illustrates the internal structure of the EDFSegment object before (a) and after the data processing steps (b)). The output record of the map function uses the channel identifier and the EDFSegment identifier as the *key* and the CSF file as the *value*, which is used as the <*key*, *value*> pair for the next phase of reduce function. The reduce function uses the channel identifier as the key to aggregate all fragments of signal data corresponding to each channel and generates a single CSF object (the details of CSF are described in the next section).

The use of MapReduce algorithm together with HDFS to implement the Cloudwave data flow has multiple advantages, including:

1. **Scalability**: The use of effective data partitioning techniques and MapReduce algorithm allows the Cloudwave data flow to leverage multiple Hadoop Data Nodes and scale with increasing volume of data.
2. **Speedup**: The parallelization of the data processing steps also allows the Cloudwave data flow to significantly reduce the total time taken to process signal data.
3. **Reliable storage and fast access**: The use of HDFS for storing the CSF files allows the Cloudwave data flow to use the HDFS data replication feature for reliable storage and parallelized read feature for fast access.

We demonstrate the scalability of the Cloudwave data flow in Section 3 (Results) using de-identified signal data generated at the CWRU EMU. In the next section, we describe the CSF that uses the epilepsy domain ontology for signal data annotation.

### The Cloudwave Signal Format (CSF) with Ontology-Based Semantic Annotation

The CSF is an extensible representation format based on the Javascript Object Model (JSON; Crockford, 1999) that is designed to address the specific data storage and analysis requirements of the epilepsy research community. In addition to epilepsy, CSF can be extended with new data and metadata fields to meet the requirements of other neuroscience and clinical research domains, for example CSF can be used to store polysomnography (PSG) data in sleep research. CSF consists of two primary information elements: (1) signal metadata; and (2) signal data, where the metadata element is divided into three sub-elements, namely: (a) study-specific metadata, (b) channel-specific metadata; and (c) clinical event annotations. Each information element in the signal metadata section uses a nested object structure of “attribute-value” pairs with arbitrary levels of nesting based on application requirements. A CSF object may be composed of additional CSF objects to support multiple-levels of granularity (e.g., all signal recording of patient over multiple visits), which is not supported in many existing signal data representation formats. CSF objects can be processed by both object model-based parsers and streaming JSON API parsers, which are now part of standard Java (EE 7) specifications (Java API for JSON Processing (JSR 353), 2014). The JSON format is similar to the eXtensible Markup Language (XML) with the flexibility to represented complex nested data but it requires significantly less space as compared to XML (Crockford, 1999).

Figure 3 illustrates an example of CSF object with signal metadata fields, epilepsy ontology annotations used to represent clinical events, and fragments of signal data. Further, the CSF object stores “structural” information (derived from the EDFSegment class) for the each fragment of signal data, including the start and end time of recording, the sequential order of each fragment, and data type of the signal (binary or integer). CSF supports random access to specific fragments of signal data (based on the associated clinical events) by using the structural information, the signal metadata, and the clinical annotation fields. The CSF object can be used to store a single EDF file or can be partitioned into smaller-sized CSF objects with a variable number of signal fragments per CSF object (similar to EDFSegment) based on the requirements of user applications or available storage resources in HDFS. The ordering of signal fragments in a CSF object is flexible, for example it can be ordered in channel-oriented order or record-oriented order (similar to EDF Data Records). The structural information in CSF keeps track of the ordering format. The CSF representation model is designed to be compatible with existing signal data representation formats, specifically the HDF5 representation format.

#### Interoperability Between CSF and Existing Signal Representation Formats

The nested representation mode of CSF is similar to the hierarchical representation model used in HDF5, which is rapidly emerging as a popular representation format for neuroscience data. It is important to note that CSF is not proposed to be a generic neuroscience data model and is designed to address specific data annotation, storage, and query requirements for epilepsy and related neurological disorders. For example, CSF supports combining random signal fragment from specific set of signal channels to construct a customized montage, which may not be required in other neurological disease domains. HDF5 consists of two primary structures namely, *groups* and *datasets*, which may have a list of *attributes* that describes user-defined information about the groups or datasets. Similar to the CSF model, the HDF5 attributes use a <*name*, *value*> structure to represent the attribute (although CSF uses this approach to represent the data objects also). The HDF5 structure stores multiple-levels of metadata information that can be used to interpret the data stored in a HDF5 file and pointers to other metadata that may include data annotations. This integrated storage of different types of metadata together with data is also similar to the approach used in CSF, which stores three categories of signal metadata together with signal data (described in the previous section).

The HDF5 specification describes three storage layout schemes to store the data on disk namely, *contiguous*, *compact*, and *chunked*. The CSF data model does not specify a data storage layout scheme as it relies on the underlying file system, such as HDFS, to store the data. The use of ontology-based terminology to annotate signal data in CSF is an important feature, which makes it easier for software applications to accurately and consistently interpret signal annotations. In contrast to traditional use of free text annotation of signal data, the ontology terms are well defined in a formal knowledge representation language. The HDF5 does not describe the use of ontology terms for data annotation, although it is possible to re-use and add CSF <*name*, *value*> pairs to HDF5. Hence, this comparison of the HDF5 and CSF structures demonstrates that CSF can be considered as a specialization of HDF5 for the epilepsy domain and it will allow the Cloudwave platform to interoperate with tools that support HDF5.

The NEO initiative is developing an object-oriented memory-based model with APIs to add and update neurological data using python libraries (Garcia et al., 2014). The NEO object model consists of 14 classes that are categorized as “data objects”, “containers”, and “grouping objects”. However, unlike CSF the NEO APIs are implemented in Python and are focused on Python libraries for generic neuroscience data. In addition, the default NEO model does not support ontology-based annotation and data partitioning, which is necessary for use in distributed computing infrastructure. Similar to HDF5, the NEO model consists of both data and metadata elements with <*key*-*value*> pairs and it clusters together data into “segments” and “blocks” (types of containers), which make it interoperable with CSF. Similar to CSF structural information, the NEO model also supports assertions of links between objects and implicit structural links between “container” and “objects”. The NEO API currently supports interoperability with HDF5, which can be extended to support CSF objects for epilepsy focused applications and parallelized data processing workflows.

#### Semantic Annotation Using Clinical Events Modeled in the Epilepsy and Seizure Ontology

A domain ontology uses formal knowledge representation language to model domain-specific terms that can be used as a standard reference terminology for annotating data and allow software tools to accurately interpret the annotations (Bodenreider and Burgun, 2009). Biomedical ontologies modeled using the description logic-based Web Ontology Language (OWL2; Hitzler et al., 2009) have been widely used for consistent annotation of data, support data integration, and enable knowledge discovery (Bodenreider and Stevens, 2006). We have developed the Epilepsy and Seizure Ontology (EpSO) as a reference terminology for epilepsy domain that can reduce terminological heterogeneity in epilepsy-focused neuroinformatics software tools. The EpSO domain ontology was developed to model multiple aspects of epilepsy, including the epilepsy syndromes, etiology, medication, seizure features, paroxysmal events, and clinical events used to annotate signal data (Sahoo et al., 2014). EpSO currently models 1350 classes with properties to represent domain-specific constraints and metadata information about the classes, which can be used by neuroinformatics tools. For example, EpSO classes are extensively annotated with free text labels describing user-friendly description of the classes, commonly used alternate labels of the class, and acronyms (e.g., “Generalized Epilepsy with Febrile Seizure Plus” is annotated with its acronym “GEFS+”).

EpSO re-uses classes from many existing biomedical ontologies and terminology systems, such as the Foundational Model of Anatomy (FMA; Rosse and Mejino, 2003), RxNorm (Nelson et al., 2011), and the Neural ElectroMagnetic Ontologies (NEMO; Dou et al., 2007). This allows EpSO to be interoperable with existing biomedical ontologies. The current version of EpSO models about twenty clinical events that are used to annotate signal data, which can be broadly divided into categories of epileptic seizures, lateralizing signs, and EEG patterns. The clinical and EEG onset/end of seizures, EEG suppression, pre-baseline and return to baseline are modeled as subclasses of *epso:EEGEvent* (*epso*: represent the EpSO namespace and resolves to the Uniform Resource Identifier (URI)).^1^ The classes describing the onset and end of specific EEG patterns, such as *epso:ContinuousSlowAcitivity* and *epso:IntermittentSlowActivity*, are modeled as subclasses of the *epso:EEGPattern*, which is also the parent class of *epso:EEGEvent*.

**Figure 4:**
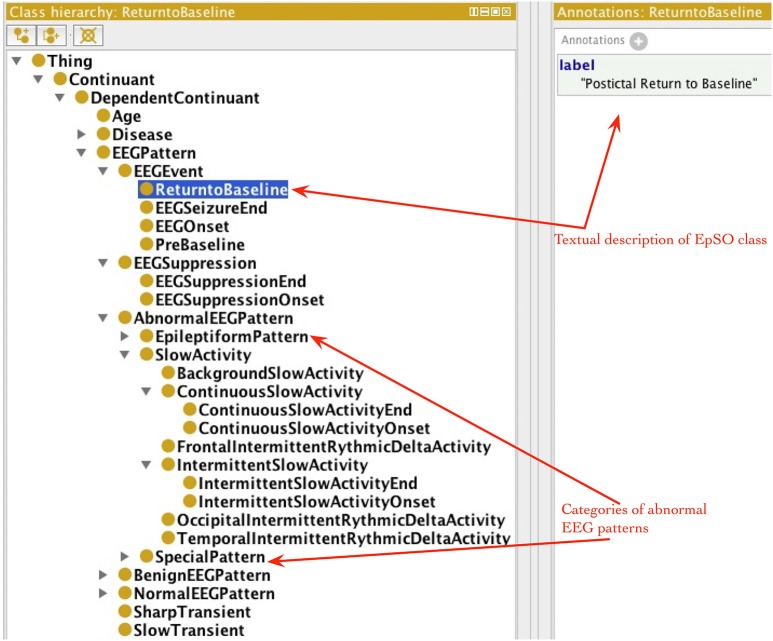
**Epilepsy and Seizure Ontology (EpSO) class hierarchy**. EpSO models 1350 classes related to epilepsy neurological disorders, including the clinical event terms used to annotate signal data. The class hierarchy of EpSO allows software application to use reasoning to improve the quality of query results and is used in cohort query user interface called Multi-Modality Epilepsy Data Capture and Integration System (MEDCIS). The EpSO classes are used as reference terminology for signal data annotation in the Cloudwave data flow, which reduces terminological heterogeneity and facilitates data sharing and integration across epilepsy informatics tools.

The occurrence of lateralizing signs (e.g., “Sign of Four”) and motor seizure events (e.g., “Clonic Seizure” and “Tonic Seizure”) are modeled as subclasses of *epso:ParoxysmalEvent* and *epso:SeizureFeature* respectively. Figure 4 illustrates a part of the EpSO class hierarchy modeling the clinical events. The EpSO class URI is used in the CSF files as signal annotations and describe the clinical events associated with the signal recordings. The EpSO class definition together with annotation properties, such as synonyms and text description (e.g., GEFS + described above), enable the Cloudwave signal visualization interface to reconcile terminological heterogeneity in signal data generated from disparate sources. EpSO can be extended to model additional neurological disorders for annotating HDF5 objects, which will ensure use of standardized terminology instead of unstructured text in neurological data annotation. In addition, the OEN ontology can be mapped to EpSO and re-use epilepsy-specific terms to facilitate interoperability of OEN compliant tools with existing EpSO compliant software tools.

### Integration of Cloudwave Data Flow with Storage and Signal Visualization Application

The primary use of the Cloudwave data flow is to support integrated processing of signal data generated from recording instruments and generation of CSF objects that can be directly used by user applications, such as the Cloudwave signal visualization module. The Cloudwave signal visualization interface is implemented in a Web browser that can be used by researchers from multiple institutions to access and visualize signal data in collaborative research projects. In contrast to existing signal visualization tools that need to be installed on individual computers, the Cloudwave visualization interface can be accessed over any Web browser (client side computation). However, a Web browser is a resource constrained platform and cannot store or process large volume of signal data (e.g., converting binary to integer format and digital to physical values), which will lead to significant delay in the response time of the visualization interface. These challenges are effectively addressed by using CSF objects (consisting of fragments of signal data) instead of EDF files. The configurable parameters of the Cloudwave data flow in terms of number of signal fragments per CSF object and duration of an epoch enable the signal visualization interface to modify the volume of signal data transferred to Web browser.

In previous work, we have demonstrated the advantages of transferring fragments of signal data corresponding to the six standard montages over the network as compared to an unpartitioned EDF file (Jayapandian et al., 2014). In addition, the CSF object stores signal data in integer format as physical values (described in Section Signal Data Processing and Flexible Data Partitioning Technique), which significantly reduces the data processing task of the Web browser. The processing of EDF files to generate channel-specific CSF objects was found to improve the response time of the Cloudwave visualization interface. In addition, the total time taken to transfer the signal data fragments with associated metadata as well as clinical event annotation and rendering of the signal data on the Web browser interface was found to be consistently less for signal data fragments (stored in CSF objects) in comparison to EDF files (Jayapandian et al., 2014). The signal visualization interface also used the annotation properties of EpSO classes to display clinical events as human readable text on the signal data. As part of our ongoing work, we are evaluating the performance of the ontology-driven query approach in the Cloudwave signal visualization module.

In addition to its use in the signal visualization application, the CSF objects generated by the Cloudwave data flow is important to address the issue of scalable storage and random access to segments of signal data. CSF objects are well suited for storage in distributed storage systems, such as HDFS, due to the storage of signal data as partitioned signal fragments. As discussed earlier, HDFS has a number of advantages as compared to traditional file systems, including ability to scale with increasing volume of data, support for multi-modal data types, and reliability through use of data replication (Shvachko et al., 2010). The Cloudwave platform can scale and reliably store the signal data as CSF objects by adding new Hadoop Data Nodes as the volume of signal data increases without disrupting the functioning of existing user application. The storage of CSF in HDFS can potentially improve the rate of data access in Cloudwave by leveraging the parallel read feature of HDFS (Shvachko et al., 2010) and we propose to evaluate this feature in our future work. In the next section, we demonstrate the scalability and performance of the Cloudwave data flow using a 30-node Hadoop cluster.

## Results

The Cloudwave data flow was evaluated to demonstrate: (a) its flexibility to support different partition schemes without adversely affecting the performance of the data flow; and (b) the scalability of the data processing algorithm by effectively leveraging Hadoop Data Nodes. The evaluation experiments were performed using de-identified signal data generated at the University Hospital Case Medical Center EMU. The data flow was executed over a High Performance Compute Cluster (HPCC) at the Case Western Reserve University (CWRU) using the open source Hadoop software (version 2.0.0). The HPCC consists of 30 data nodes and a master node that are connected by a 10 Gigabit Ethernet (GigE). The master node has a dual quad-core Intel Xeon 5150 2.66 GHz processor and the data nodes have dual quad-core Intel Xeon 5450 3.0 GHz processors with 16 GB of memory each. The HPCC is within the CWRU firewall, which allowed the use of de-identified patient data for evaluating the Cloudwave Data Flow. Due to space limitation on the individual data nodes of the HPCC, the maximum volume of signal data used in the experiment is 25 GB, which included the clinical event annotation and signal metadata.

### Performance of Cloudwave Data Flow with Variable-Sized Signal Data Fragments

The support for signal data partitioning is an important feature of the Cloudwave data flow that allows it to process large EDF files using Hadoop Data Nodes with limited memory resources (described earlier in Section Signal Data Processing and Flexible Data Partitioning Technique). This evaluation is to validate the hypothesis that the number of data fragments per EDFSegment object can be flexibly changed without affecting the overall performance of the Cloudwave data flow. This flexibility of the data partitioning approach is important to allow the data flow to be deployed on different types of Hadoop Data Nodes. We evaluated the effect of different number of fragments per EDFSegment object on the Cloudwave data flow by partitioning 25 GB of signal data and using two configurations of 15 and 30 Hadoop Data Nodes in the HPCC. Each fragment of 30 s epoch corresponds to 0.648 MB of signal data (in binary format) and the number of fragments per EDFSegment object was increased (from 2 to 16 fragments) until the available memory in the Hadoop Data Nodes was exhausted during the evaluation.

**Figure 5:**
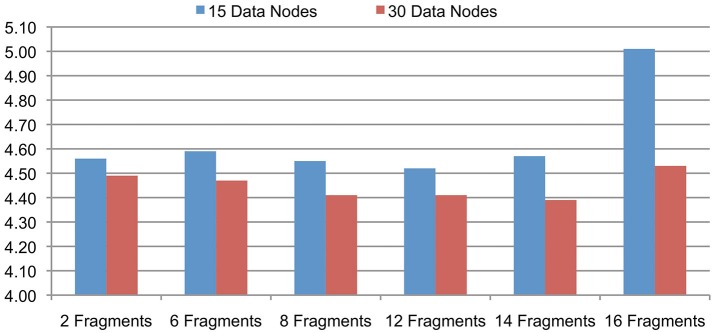
**Cloudwave data flow evaluation results with variable-sized signal data fragments**. The number of signal data fragments in an EDFSegment object can be modified according to available memory in the Hadoop Data Nodes. The results of this experiment demonstrate that for 25 GB of EDF files processed on 15 and 30 Data Nodes, the change in total number of fragments per EDFSegment does not lead to significant variations in performance of the Cloudwave data flow. This parameter can be tuned to get maximum improvement in performance of the Cloudwave data flow, for example 12 and 14 signal fragments per EDFSegment object are optimal values for 15 and 30 Hadoop Data Nodes respectively.

Figure 5 shows that the performance of the Cloudwave data flow does not vary significantly with increase in the number of signal data fragments per EDFSegment object for both the 15 and 30 Data Node configurations. The reported results are an average of three consecutive runs with the first run executed on a cold cache. The results demonstrate the Cloudwave data flow can be configured to use the maximum available memory on a Hadoop Data Node without affecting its performance. At present, the configuration parameter is modified manually, however in future we propose to enable the Cloudwave data flow to dynamically adjust the fragment per EDFSegment parameter by using an error logging mechanism. The results show that the available memory on the CWRU HPCC Data Nodes supported a maximum of 16 signal data fragments (10.94 MB) per EDFSegment object (although 14 data fragments give better performance results). The results also demonstrate that the time taken to process the data is lower for the 30 nodes configuration as compared to the 15 nodes configuration, which shows that the data flow effectively parallelizes the computations to leverage available Hadoop Data Nodes. In the next section, we describe a more detailed evaluation to demonstrate the scalability of the Cloudwave data flow.

### Scalability of The Cloudwave Data Flow

We evaluate the scalability of the Cloudwave data flow in terms of: (a) ability to process increasing volume of signal data with corresponding change in total time; and (b) ability to leverage increasing number of Hadoop Data Nodes to reduce the total data processing time for fixed volume of signal data. Seven datasets of EDF files with sizes ranging from 100 MB to 25 GB were created and the complete Cloudwave data flow was executed during the experiment. Using the Cloudwave partitioning techniques, two categories of the seven datasets were generated with 8 and 16 fragments per EDFSegment object. These 14 datasets were processed using six configurations of Hadoop Data Nodes ranging from 1 to 30 Data Nodes to create CSF data objects, each with 8 and 16 signal fragments. Each combination of dataset and Data Node configurations (14 datasets and 6 Data Node configurations) was executed for three consecutive runs (starting with a cold cache) and the average values are reported.

**Figure 6:**
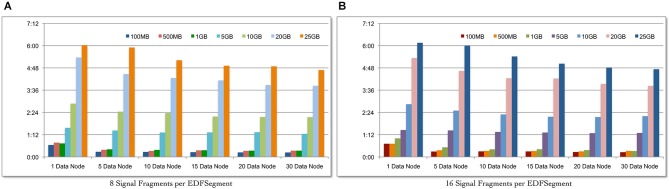
**Scalability of the Cloudwave data flow with increasing size of data**. The Cloudwave data flow effectively uses multiple Hadoop Data Nodes to scale with increasing amount of data and consistently reduces the total time taken to process the data. The results also demonstrate that the data partitioning approach allows the Cloudwave data flow to flexibly modify the volume of signal data per EDFSegment (total number of signal fragments) without adversely affecting time performance (EDFSegments with 8 **(A)** and 16 **(B)** fragments have comparable performance).

Figure 6A shows that the Cloudwave data flow scales with increasing volume of signal data (with 8 signal fragments per EDFSegment object) and effectively leverages the increasing number of Hadoop Data Nodes to significantly reduce the total data processing time. Figure 6B shows similar results for 16 signal data fragments per EDFSegment object, which is consistent with previous results that showed that changes in number of fragments does not affect the performance of the data flow (Section Performance of Cloudwave Data Flow with Variable-sized Signal Data Fragments). The increase in Hadoop Data Nodes from 1 to 30 improves the performance of the data flow by 64.2% for 100 MB of data with 16 fragments per EDFSegment object (Figure 6B) and by 63.15% with 8 fragments per EDFSegment object (Figure 6A). The performance of the data flow improves by smaller percentage of 27.2% for 25 GB of data with 16 fragments per EDFSegment object (and 26.6% for 8 fragments per EDFSegment object, Figure 6A). We are exploring additional approaches to use greater parallelization to improve the performance of the data flow for larger sizes of signal data.

The improvement in performance of the data flow with increase in parallelization is clear since the total data processing time decreases as the number of Hadoop Data Nodes is increased for the larger signal datasets. For example, as the number of Data Nodes is doubled from 5 to 10 and from 10 to 20 for 25 GB of data (with 16 fragments per EDFSegment), the performance of the data flow improves by 12.5% and 14.4% respectively. However, there is no improvement in performance of the data flow as the number of Data Nodes is doubled from 5 to 10 and negligible improvement of 0.02 s for increase in Data Nodes from 10 to 20 for 100 MB data (with 16 fragments per EDFSegment). We are analyzing our current algorithm to address this issue. It is interesting to note that there is an order of magnitude difference between the rate of increase in data size (from 100 MB to 25 GB) and the rate of increase in data processing time (from approximately 15 s to 4.4 min). This slower increase in data processing time (as compared to increase in volume of data) can be further improved with more effective parallelization approaches, which is part of our ongoing work in the Cloudwave project.

## Discussion

The increasing complexity of neuroscience data and especially electrophysiological signal Big Data has made it difficult to manage data using traditional informatics infrastructure that use existing database models (e.g., relational database) to store and retrieve data (Mouček et al., 2014). In addition to storage, there is an important requirement to develop scalable neurosciences data processing approaches that can take advantage of parallel and distributed computing techniques for large volume of data that is generated at a high velocity. The Cloudwave data flow is designed to meet these two requirements and uses EpSO to address the issue of terminological heterogeneity to facilitate data sharing and integration. The primary features of the Cloudwave data flow include the use of Hadoop MapReduce and HDFS together with the flexibility to configure multiple parameters based on the availability of resources on a Hadoop cluster. This allows Cloudwave data flow to be deployed on different types of Hadoop clusters and to be used as a template to develop scalable neuroscience data processing data flow in many existing neuroinformatics projects, such as the GNDataPlatform (Sobolev et al., 2014a).

Similarly, the Cloudwave data flow can be integrated with existing large data linking and sharing initiatives in neuroscience, such as the INCF Dataspace and the International Epilepsy Electrophysiology portal (IEEG; Wagenaar et al., 2013), for high performance data processing and analysis. The INCF dataspace may offer the Cloudwave data flow as a service by using the Software as a Service (SaaS) approach, which will allow users to process signal data using the instances of Cloudwave data flow hosted by INCF to generate HDF5 or CSF data objects. This service will significantly reduce the computational requirements for users and support standard-based signal data sharing. The IEEG portal is a large U.S National Institutes of Health (NIH) project that stores signal data in the cloud and provides Matlab-based tools to analyze the data (IEEG-Portal). The IEEG portal uses the Multiscale Electrophysiology Format (MEF; Brinkmann et al., 2009), which uses data compression, encryption, and cyclic redundancy check for identifying data errors, to store the data in cloud. At present, the IEEG-Portal supports the download of datasets from the Amazon Web Services (AWS) cloud platform and subsequent analysis using Matlab tools (Ieeg-Portal, 2014). The integration of the Cloudwave data flow with the IEEG-Portal will allow greater support for Java-based signal data analysis tools and use of EpSO classes for signal data annotation. The ontology-based signal annotation will significantly improve the query feature of the IEEG portal for users.

The use of EpSO as an epilepsy domain ontology is a novel feature of the Cloudwave platform. In addition to its role in reducing terminological heterogeneity in signal data annotation, it is also being used to support constructing patient cohort queries in the PRISM project for clinical research in epilepsy (Sahoo et al., 2014). A similar functionality to support querying of signal data based on clinical event annotation, study metadata, and signal-specific metadata is under development in the Cloudwave project. The use of EpSO for querying signal data will allow use of description logic-based reasoning to improve the quality of results. For example, existing approaches to query for signal segments annotated with interictal events cannot select signal data annotated with “spike” although “spike” is a sub category of interictal event, which is explicitly modeled in EpSO. Hence, use of EpSO for signal data annotation (implemented in the Cloudwave data flow) and querying will address the limitations of lexical matching-based query execution techniques. In addition, the use of EpSO to model signal montages can be used to pre-compute these values in Cloudwave platform using channel-specific signal fragments from CSF objects. The pre-computed values can be stored as CSF objects in HDFS and transferred to the signal visualization module to reduce computational time and improve responsiveness of the user interface.

## Conclusion

The paper describes the development of a MapReduce-based high performance scalable electrophysiological signal processing data flow, which was developed as part of the Cloudwave project to address the challenges of volume and velocity of signal data. The Cloudwave data flow processes one or more EDF files to generate CSF data objects, which is an extensible JSON-based signal data representation format, with partitioned fragments of signal data for storage and processing in HDFS. The CSF model is compatible with existing neuroscience data representation formats, such as HDF5 and NEO object-oriented APIs, with ontology-based signal annotations to address terminological heterogeneity in neuroinformatics tools. The evaluation of the Cloudwave data flow on a 30-node Hadoop Data Nodes validate the effectiveness of using MapReduce algorithm to scale with increasing volume of signal data. The Cloudwave data flow not only meets the requirements of user applications such as signal visualization, but it can also be integrated with existing large neuroscience data repositories such as INCF dataspace and IEEG-Portal.

## Authors and Contributors

CJ, AW, and SSS developed the data partitioning technique, MapReduce algorithm, and CSF model. SSS designed the evaluation experiments that were implemented by CJ, AW, and PR. SDL, KL, GQZ, and SSS designed the Cloudwave platform with features for epilepsy clinical research, signal visualization, and filtering functions. SDL, BG, and KL validated the features of signal visualization interface. All authors contributed to preparation of the paper, figures, and charts.

## Conflict of Interest Statement

The authors declare that the research was conducted in the absence of any commercial or financial relationships that could be construed as a potential conflict of interest.

## Footnotes

^1^http://www.case.edu/EpSO.owl
